# Intelligent robotics harvesting system process for fruits grasping prediction

**DOI:** 10.1038/s41598-024-52743-8

**Published:** 2024-02-03

**Authors:** K. M. Alaaudeen, Shitharth Selvarajan, Hariprasath Manoharan, Rutvij H. Jhaveri

**Affiliations:** 1 Department of Computer Science and Engineering, Grace College of Engineering, Mullakkadu, Thoothukoodi, India; 2 School of Built Environment, Engineering and Computing, Leeds Beckett University, LS1 3HE Leeds, UK; 3 Department of Computer Science, Kebri Dehar University, Kebri Dehar, Ethiopia; 4 Department of Electronics and Communication Engineering, Panimalar Engineering College, Poonamallee, Chennai, Tamil Nadu India; 5 Department of Computer Science and Engineering, School of Technology, Pandit Deendayal Energy University, Gandhinagar, India

**Keywords:** Engineering, Electrical and electronic engineering

## Abstract

This paper proposes and executes an in-depth learning-based image processing approach for self-picking apples. The system includes a lightweight one-step detection network for fruit recognition. As well as computer vision to analyze the point class and anticipate a correct approach position for each fruit before grabbing. Using the raw inputs from a high-resolution camera, fruit recognition and instance segmentation are done on RGB photos. The computer vision classification and grasping systems are integrated and outcomes from tree-grown foods are provided as input information and output methodology poses for every apple and orange to robotic arm execution. Before RGB picture data is acquired from laboratory and plantation environments, the developed vision method will be evaluated. Robot harvest experiment is conducted in indoor as well as outdoor to evaluate the proposed harvesting system's performance. The research findings suggest that the proposed vision technique can control robotic harvesting effectively and precisely where the success rate of identification is increased above 95% in case of post prediction process with reattempts of less than 12%.

## Introduction

As the entire society is moving towards autonomous operations it is necessary to carry out different application operations such as military, civil aviation, medical applications, transportations etc. with the help of robotic systems. It is observed that in recent time most of the robotic systems are currently deployed in the process of agriculture as a replacement of humans as it is considered as time consuming process. In the process of Internet of Things (IoT) where multiple sensors are used in the detection process the agricultural lands will be surveyed during crop harvesting thereby appropriate crops will be planted and monitoring status is defined in an exact way. As a replacement of IoT the proposed method considers robotic system which is connected with multiple sensors for monitoring states. The major advantage of the replacement process is that the robotic systems can move in all different directions and it will analyze the entire area of agricultural land by using a computer vision procedure. In addition all the obstacles that are present in the mid-way is identified and changes are provided for processing autonomous operations by using a fixed camera at the front end of each robot. The above mentioned process is carried out at higher speed and the exact solution for plantation is provided by considering the depth of enclosures. Further the designed robot can able to identify the amount of water that is present in the agricultural land and the time period of yield will be determined in such cases and the outcomes of monitoring states is transmitted to corresponding individuals within short period of time.

**Figure 1:**
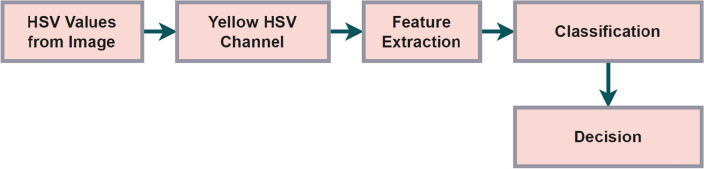
Robotic decision development with image processing units.

Figure 1 portrays that images are captured at the front end of robotic systems where a High Standard Value (HSV) is grasped from image set that is present under same agricultural area. The HSV values are used for determining the possibility of initial state conditions where a yellow HSV channel is connected and entire feature of agricultural lands are extracted. Further every image is classified according to necessary contents and the robot provides an automated decision based on the current condition of crops or other types of plantations. Once the plantation is processed then the robotic systems can able to recognize different types of fruits and it will pick up the necessary fruit without any obstacles..

### Background and related works

In the mid-1980s, software engineering was utilized to enhance farming management practices and provide new advancements. Horticultural advancements are progressing in the recently introduced paradigm of Agriculture 4.0^1^. The executives in Agriculture 4.0 combine emerging innovations such as mechanical technology, the Internet of Things (IoT), machine vision, and artificial intelligence with a focus on economic yield. Agriculture 4.0^2^ can be seen as an extension of the Industry 4.0 vision, specifically tailored for the agricultural sector. Agricultural cyber-physical systems (CPS)^3,4^, along with agricultural IoT, have recently garnered significant interest. Actuators and sensors that interact with the physical environment can be utilized to intelligently and automatically control crop growth settings. The issues confronting the development of CPS^5,6^ include real-time processing, adaptability, hardware and software code verification, and scalability. Fruit recognition is a critical challenge in autonomous agricultural applications. Traditional approaches involve the utilisation of manually designed characteristics to represent the objects, followed by the application of machine learning techniques to identify or separate an object based on these extracted characteristics. Agricultural cyber-physical systems (CPS) have recently gained significant attention, surpassing the focus on Internet of Things (IoT) in farming^7,8^. Therefore, it is possible to control intelligent and automated crop development settings by interacting with real environmental variables using sensors and actuators. The development of CPS will encounter various problems, such as the need for continuous planning, adaptability, coordination of equipment programming, and diversity^9–11^. Exploration often centers around the development of intelligent agricultural robots that can efficiently operate on a large scale, especially those with practical material capabilities. Agrobots have been deployed to this location for precise horticultural tasks such as weed removal, harvesting, irrigation, and maintenance. An innovative concept for a robotic system designed specifically for harvesting cherry tomatoes is presented. The robot system comprises a railed vehicle, a stereo vision device, a fruit collector, a manipulator, and an end effector. An automated kiwifruit harvesting robot design has been documented.

In^12^, the robot utilizes a Global-Positioning System (GPS) and machine vision to adhere to radio commands and autonomously explore; the structure consists of four picking arms that are controlled by a single centralized handling unit. A ground robot, equipped with a camera, was utilized alongside a small dosage framework to develop a compact and precise herbicide administration system^13^. Another artwork portrayed a self-governing robot specifically engineered for the purpose of harvesting apples. The system comprises three primary elements: a mobility apparatus, a visual system, and a mechanical arm outfitted with a gripper. The current architectural design includes a comprehensive system that consists of an RGB-D camera, a gripper, and a mechanical arm^14^. These components are smoothly incorporated into an autonomous wheeled robot. The mechanized weeding versatile stage provides three mechanical implements for weed control, namely the bolt cultivator, the prong, and the cutting tool. The choice of the suitable gadget relies on the particular weed that has been identified. The dominant pattern seen in the current literature is on the prevalence of mobile robots that are specifically developed and optimized for particular tasks. Multiple agricultural robots are currently available on the market, each designed for certain agricultural tasks. There exists a robot named Harvest that is specifically engineered for the purpose of harvesting strawberries^15^. Another robot, named Guss, is employed for the purpose of orchard spraying. In addition, there are agricultural robots named Oz, Ted, and Dino that are specifically utilized for the task of removing weeds^16^. The Digital Farmhand, Farm droid, and Clear Path14 are examples of commercially accessible versatile robotic platforms specifically engineered to carry out diverse agricultural functions. Collecting sensor data is a crucial component of computer hardware architecture. Indeed, greenhouses are not employed for cultivating all types of crops. The planting habitats of certain crops, such as dragon fruits, are undergoing expansion and modification. Cameras are strategically placed in designated regions inside agricultural systems to capture images of crop growth, enabling the monitoring of crop development. Nevertheless, this method of image capture restricts the comprehensiveness of recording crop growth images, as it can only follow a limited number of crop sides. The number of sensors used increases directly in relation to the size of the farm. Another method in recent years has been to employ robot movement to interact with sensors15. Data collection through the utilisation of sensors is highly versatile and efficient, thereby leading to potential cost reduction in the installation of sensor networks. Furthermore, certain researchers16 devised the robots by employing integrated convolutional neural networks (CNNs) to identify certain crops for subsequent processing, hence enabling accurate documentation of crop growth images. Defoliation, lateral branch reduction, and green harvest are widely recognized viticulture procedures that significantly influence grape quality^17^. Activities such as canopy management, pre-harvest, post-harvest, and harvest are considered to have a beneficial effect on the quality of wine in valuable crops such as wine grapes. Regarding high-value crops such as wine grapes, it is believed that the management of shelter, pre-harvest, post-harvest, and storage practices have a significant impact on the quality of wine. Agrobots are designed to enhance viniculture by reducing the need for human labor through the execution of viticulture tasks with the proficiency of a skilled professional^18^.

### Major contributions

To create a fully operational robotic harvesting system, there are still a lot of obstacles to overcome. Therefore this paper offers a yield scale multi-reason autonomous grape gatherer robot, or ARG for short, that is designed to do viticulture tasks such as collect, green reap, and defoliation instead of a skilled specialist. In practical implementation settings, traditional vision methods are susceptible to mistakes in precision, resilience, and efficiency. The development and evaluation of an automated apple harvesting vision approach using deep learning is the focus of this work. In this paper, the following contributions are highlighted:

- A computationally efficient one-stage instance segmentation procedure for conducting fruit identification and instance segmentation on sensory input is proposed using computer vision techniques.
- Using edge and contour points from an RGB camera, propose an enhanced computer vision-based network for ripe fruit detection and grasping estimate.
- System integrates and combines the above two functions while designing and implementing an accurate robotic system for autonomous fruit harvesting

### Paper organization

The remainder of this document is laid out as follows: The second section looks at studies on fruit recognition and grasping estimation that is comparable to the first. “Materials and methods” section introduces the suggested visual processing algorithm's techniques. The experimental setup and results are presented in “Results” section. “Conclusions” section concludes the research work with future updates.

## Materials and methods

**Figure 2:**
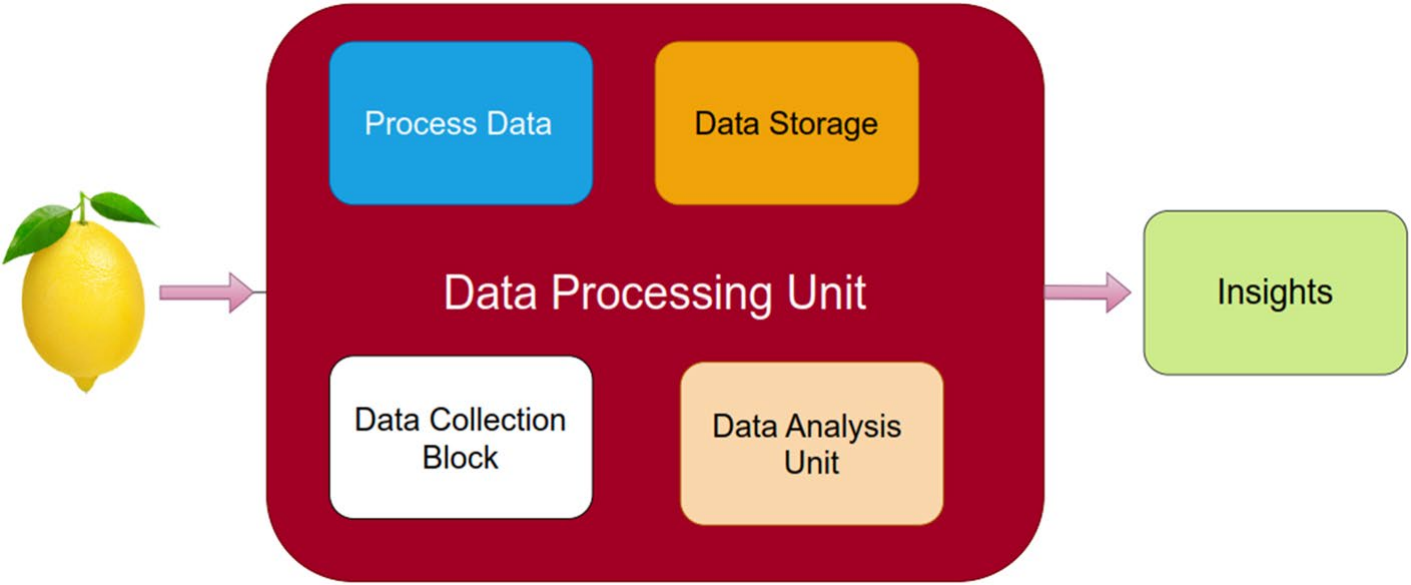
Proposed architecture of intelligent robotics harvesting system process for fruits grasping prediction.

The mobile base is a special mobile vehicle mainly composed of a main control unit, four electric wheels, a 24 V power supply and a vehicle chassis. The entire robotic system, including the movable base, is programmed to find the correct position. Intelligent Robotics is a robotic arm with 6 degrees of freedom. Along with end effectors, manipulators support path development. Our end-effectors are built using soft robotic grippers that have been extensively studied for robotic grippers. The secure touch and the yielding mechanism are integrated in the suggested end effectors as the consequence of ribs and low modulus material. The Real Sense RGBD camera is a major component of the visual subsystem. This is used for fruit photography for processing the upcoming data. The collected fruit position and alignment data is used to control robotic reaping equipment. Figure 2 shows the overall operation of the robotic harvesting system.

**Figure 3:**
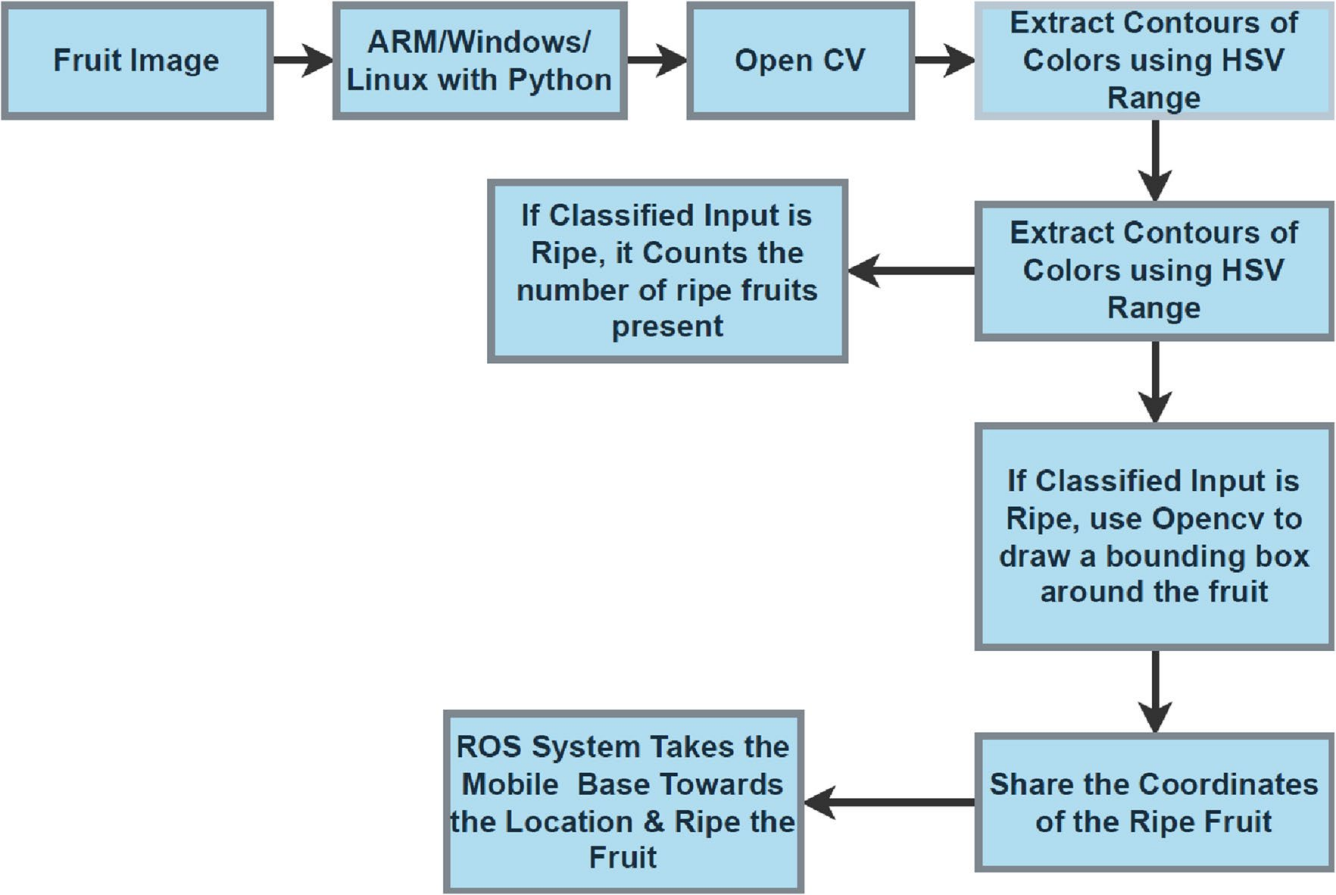
Proposed apple harvesting robot software designs.

The two phases in the our vision technique are fruit identification and grabbing assessment. The RGB images are segmented and detected by the suggested vision algorithm in its first step. Contours are created by mixing the anticipated mask of every fruit with the depth image from Open CV's (Open Computer Visions), input point clouds. Contours, using Open CV will anticipate the form, size, and approaching attitude of each fruit based on the output from the first phase in the second step. Figure 3 provides step-by-step processing for apple harvesting robotic systems.

### Network architecture

To perform fruit identification, consider the lemon embed with this study uses computer vision and HSV channels to create an advanced, lightweight, one-step, Fully Convolutional detection network called "Single Shot Detection (SSD)". Instead of using resnet50 as the backbone network and the previous network using a three-layer pyramidal network of features, the proposed classification based on SSD HSV uses simplified “computer vision” backbone and HSV values. On a 128-core GPU based on NVIDIA Maxwell™ architecture, the proposed SSD weighs 20.5 MB and averages 30 frames per second. Each classification level has an instance split branch and an edge coordinate detection block. To analyze data at multiple scales, computer vision, scaled convolutions with different aspect ratios are often employed to detect objects of various shapes and sizes within an image. These convolutions, often utilized in object detection frameworks like Single Shot Multibox Detector (SSD) or You Only Look Once (YOLO), involve using convolutional kernels of different aspect ratios (typically rectangles with various width-to-height ratios) to capture objects with diverse shapes and uses scaled convolutions with different ratios (eg 1, 2, 4). Segmentation can be classified into two as follows: segmentation by mask and segmentation by detection. The confidence score, bounding box and class of the mesh elements are predicted by the detection segmentation method. Each classification level uses a bounding box which has a preset value of 50 × 50 at C4 level and 120 × 120 at C5 level respectively. The process of segmentation by binary branch is based on the SPR Net architecture, which allows you to predict binary masks for features in a feature map on a pixel-by-pixel basis. Although not used in this work, semantic branching in computer vision methods is also designed for semantic branching.

**Figure 4:**
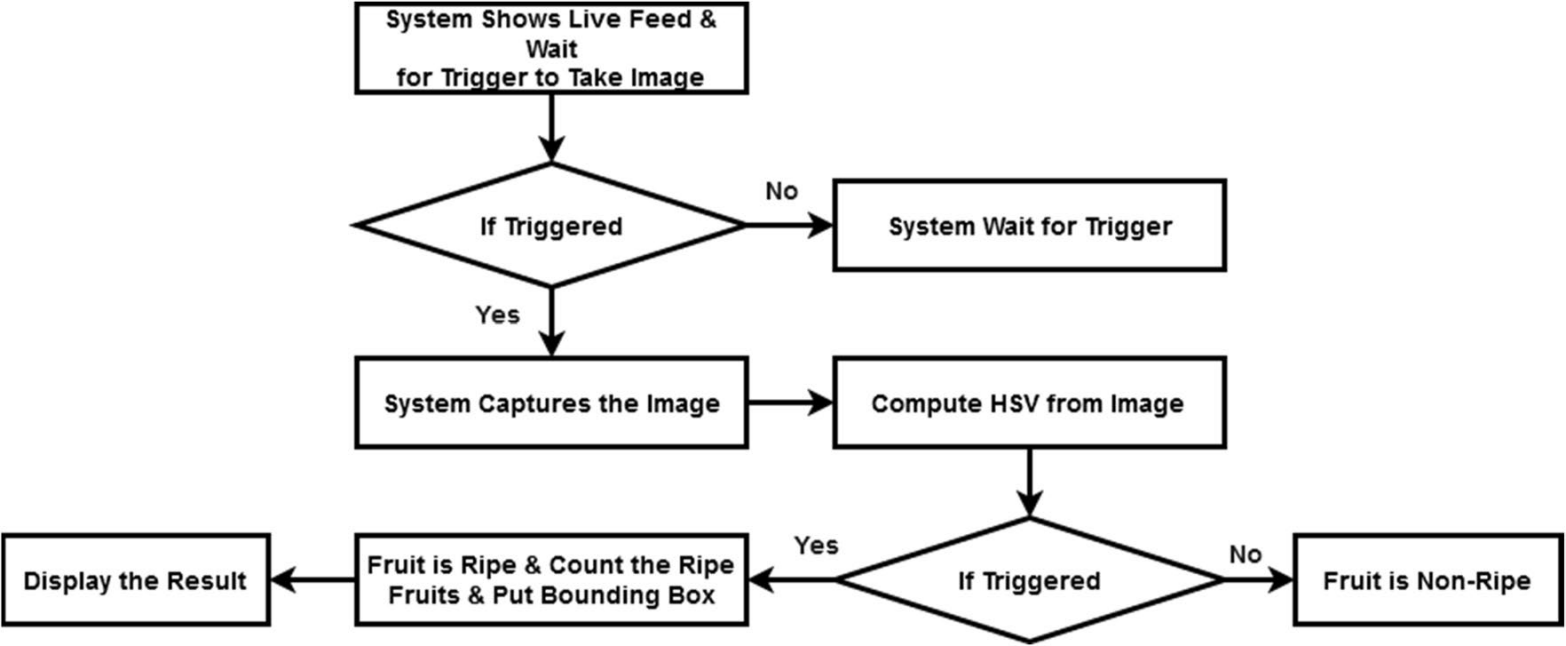
Fruit classification architecture.

To increase the versatility of the classification test, we collected 1200 photos from different contexts. For example, farmlands in China, Melbourne and Australia have different settings and lighting, including artificial lighting, shadows, daylight, lighting in front, side and backlighting. Label images were used to confirm classification. HSV correction uses reflection (horizontal only), random saturation (0.8–1.2), scaling (0.8–1.2), rotation (10), and brightness (0.8–1.2). A high-definition camera collects images of the real world with a computer vision process. Computer vision supports conversion from RGB to HSV. With the help of HSV, the values are sent to a specific channel responsible for feature extraction of the classification system according to the selected threshold. When the threshold is exceeded, the program creates a bounding box and displays the result as shown in Fig. 4. This is the first part included in the data acquisition system to inject data into the ROS system for data collection.

### Grasping estimation

This graphic depicts an apple as a spherical shape. In natural circumstances, apples can be blocked from the RGB-D camera's view-angle. As a result, the visible area of the apple from the RGB-D camera's current view-angle shows the robotic arm approaching the target in the correct location. As an object pose estimation problem, we employ Contours with Open CV to provide grasping estimates. From the current view angle, as an approaching posture, a vector from the geometric center to the visible center of the apple is selected. Our system can predict the approaching posture using only a single-viewed point cloud as input, decreasing the operation time in half and the dynamics model of prosthetic can be established. The equation is shown as follow.

1 $$Mq+H\left(q,q\right)={\gamma }^{i}-JF-cq- {k}_{i}q-{T}_{k}$$

where M represent the Matrix ,$H\left(q,q\right)$ a need to pick the object for grasping mechanism, ${\gamma }^{i}$ denotes the torque used for the motion, F denoted the external force, ${k}_{i}q$ need for restoring point and ${T}_{k}$ initial condition of the robot.

### Contours using open CV architecture

**Figure 5:**
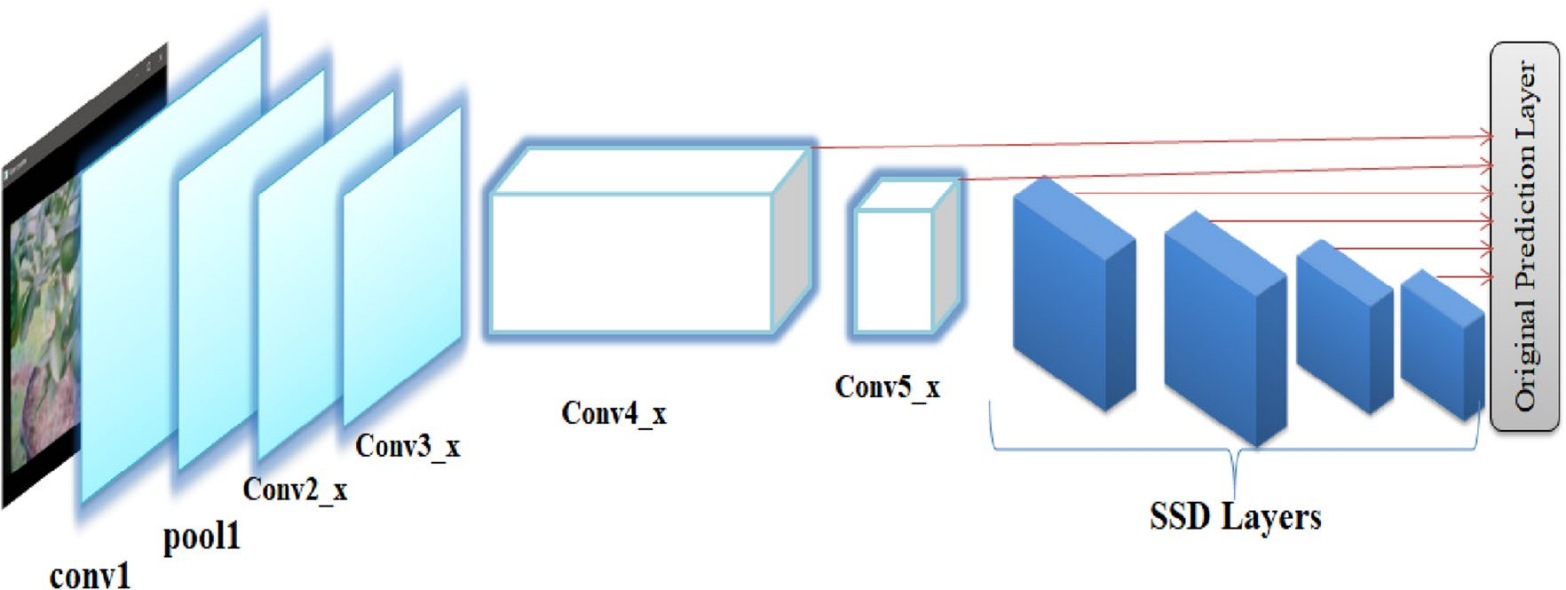
SSD architecture for fruits grasping prediction.

In this work, maybe already familiar with the word contour. This term has been mentioned several times in previous posts. A contour line is a curved line that represents the threshold between two sets of values or intensities. Because the two terms are frequently used interchangeably, it might be perplexing. Simply expressed, the concept of edges exists in a local range, but the concept of contours exists at overall border as shown in Fig. 5. Edges are points where the values differ greatly from those of their neighbors. Contours, on the other hand, are closed curves formed by edges and representing a figure's boundaries. The contour can be used for a wide range of purposes. With the help of the concept of image moment, we may locate the centroid of a picture or calculate the area of a boundary field. In popular usage, a ‘moment' refers to a brief period. However, in physics, a moment is defined as the product of a distance and another physical quantity, which refers to how a physical quantity is dispersed or positioned. Image moment describes how image pixel intensities are dispersed according to their position in computer vision. We can derive the centroid or spatial information from the picture moment, which is a weighted average of image pixel intensities.

2 $${\text{x}} = {\text{M}}\_{1}0/{\text{M}}\_00$$

3 $${\text{y }} = {\text{M}}\_0{1}/{\text{M}}\_00$$

In the X-axis, the variable 'M' transitions from the positive to the negative axis initially, while in the Y-axis, it ranges from the negative to the positive axis in relation to the zero axis.

## Results

A high definition camera helps us to collect the real-time feed from the field from the image our computer vision techniques extracts the HSV(Hue Saturation Value) value classifies the fruit as ripe or non-ripe and share a contour, edge, and center co-ordinate to the moving vehicle which is basically a manipulator for industrial robotics a customized soft end-effector for an intelligent robot Logitech 1080UHD USB web camera, and an ARM Cortex-A53 1.4GHz central control computer make up the designed robotic harvesting system. With the help of HSV, it transfers the value to the particular channel that the channel takes care of the feature extraction for the classification system. The classification system classifies and decided to rip the fruit or not as shown in Fig. 1. If it is ripe fruit then the system sends the coordinates to ripe the fruit to the control system. The control system is built using Linux Ubuntu 18.04 and the Robot Operating System (ROS) in Melodic along with the mobile robot unit. ROS and Gazebo handle communication between the RGB camera and the computer which takes care of the computer vision process of classification. To evaluate the laboratory and real time outcomes for designed robotic systems the following case studies are considered.

Case study 1: Fruit form estimation.
Case study 2: Accuracy of grasping.
Case study 3: Robotic hold conditions.

All the case studies are performed with image classification technique using python coding and the sample code is provided as follows.

The unique deep learning-based technique was compared to two old methods, sphere Random Sample Consensus (sphere-RANSAC) and sphere Hough Transform (sphere-HT). The RANSAC and HT techniques both employed point clouds to estimate the form of the fruit. RGB-D pictures from both laboratory and orchard contexts were used to do this comparison. We included a thick clutter condition in the experiment to see how well the algorithm performed when the fruits were close together.

### Case study 1

**Figure 6:**
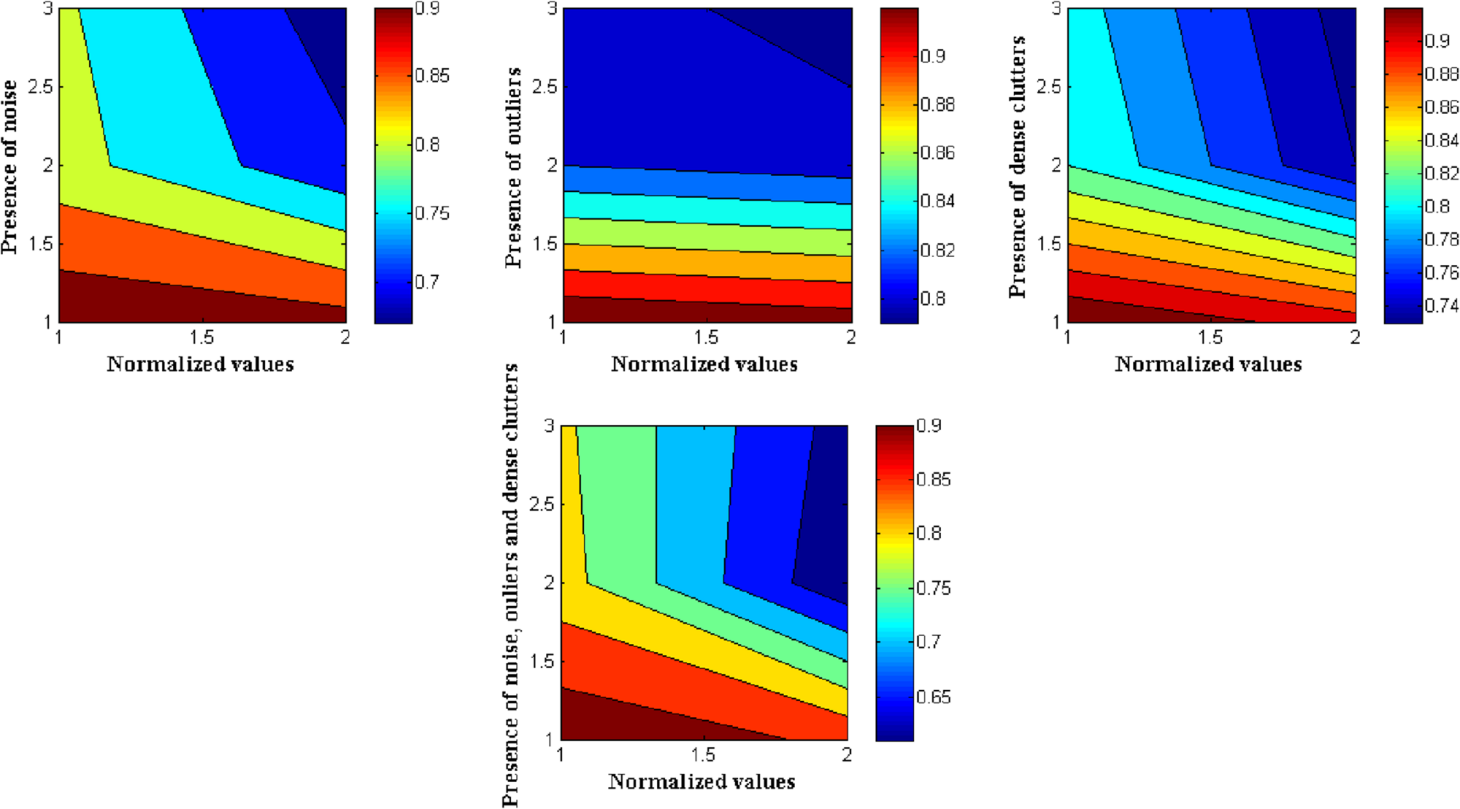
Comparison of different activities with normalized values.


Figure 6 shows the outcomes of a variety of tactics in various settings. Contours using an Open CV-based technique greatly improves the localization accuracy of the 2D bounding box (0.94 in typical conditions), outlier (1% to 5% of the aggregate of point clouds), and point clouds to assess the durability of various strategies in dealing with noisy and unexpected conditions When dealing with outliers, three strategies showed comparable robustness. Both RANSAC and HT employed a voting system to estimate the shape's primitives, which proved to be resilient against outliers. When confronted with noisy data, Contours employing Open CV-based approaches displayed far stronger resilience, with just a 3% loss in outcomes when compared to the usual scenario, whereas exhibited substantial decreases in accuracy compared to the Contours using Open CV. Contours utilizing Open CV outperformed the other two strategies in the dense clutter case when compared to the other two techniques. When compared to existing approaches, the experimental findings show that the Contours using the Open CV-based method enhances grasping estimating accuracy and resilience.

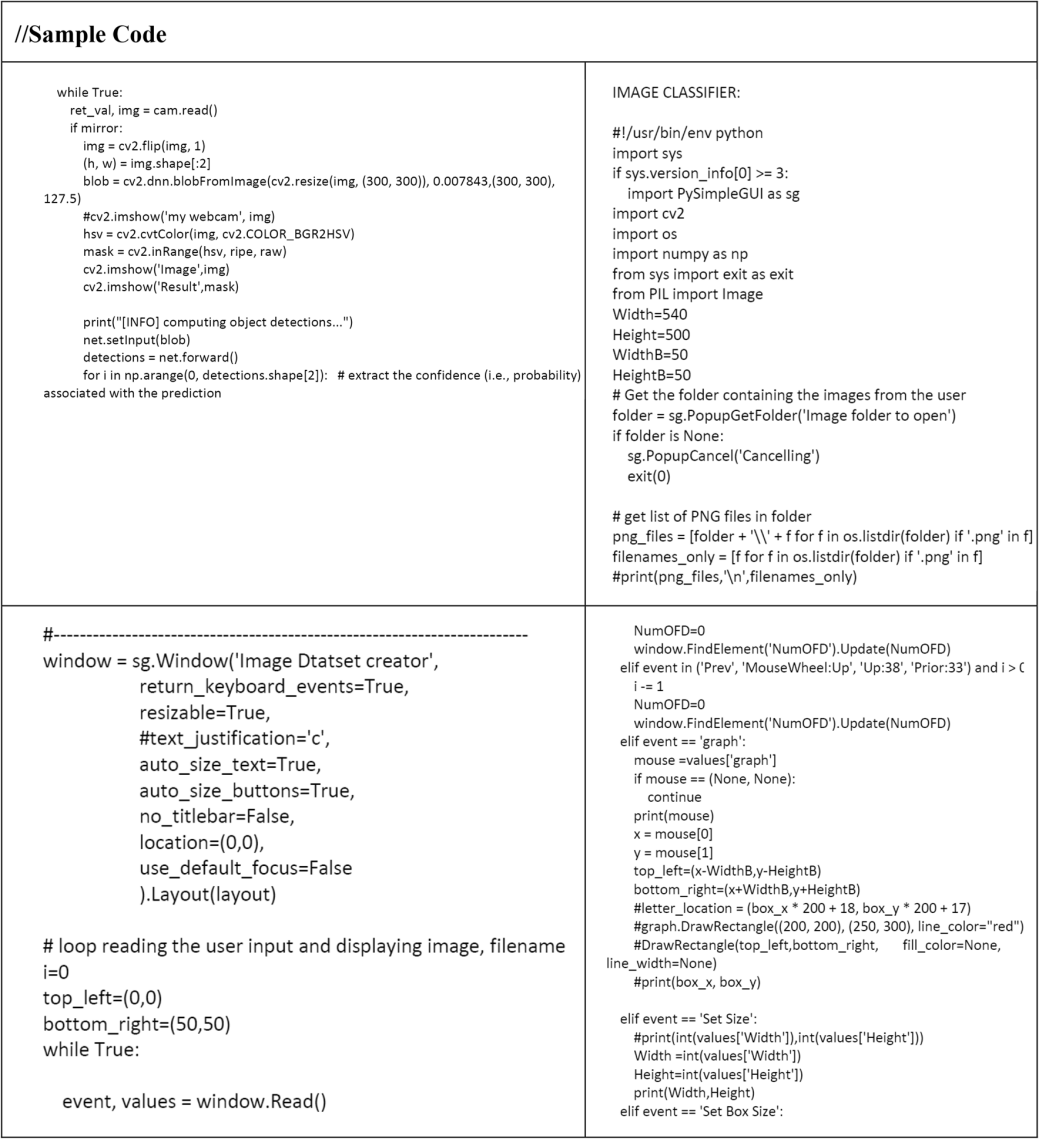


The experimental results for approaching posture prediction revealed that the Contours using the Open CV-based technique performed well. Experiments showed that Contours using Open CV grasping estimates can detect object grip orientation effectively and robustly in noisy, outlier-presented, and high-clutter settings. The key functions in explainable codes are provided as follows.
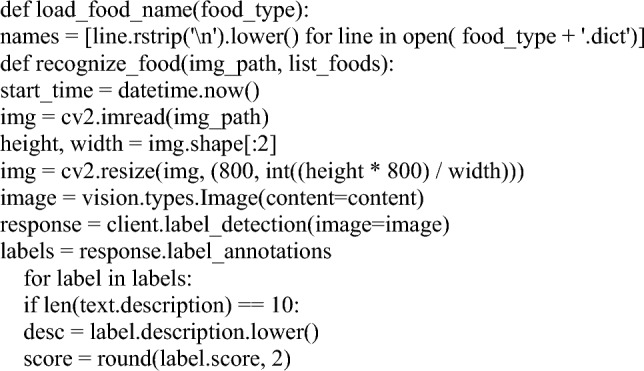


The experimental results for approaching posture prediction revealed that the Contours using the Open CV-based technique performed well. Experiments showed that Contours using Open CV grasping estimates can detect object grip orientation effectively and robustly in noisy, outlier-presented, and high-clutter settings.

### Case study 2

**Figure 7:**
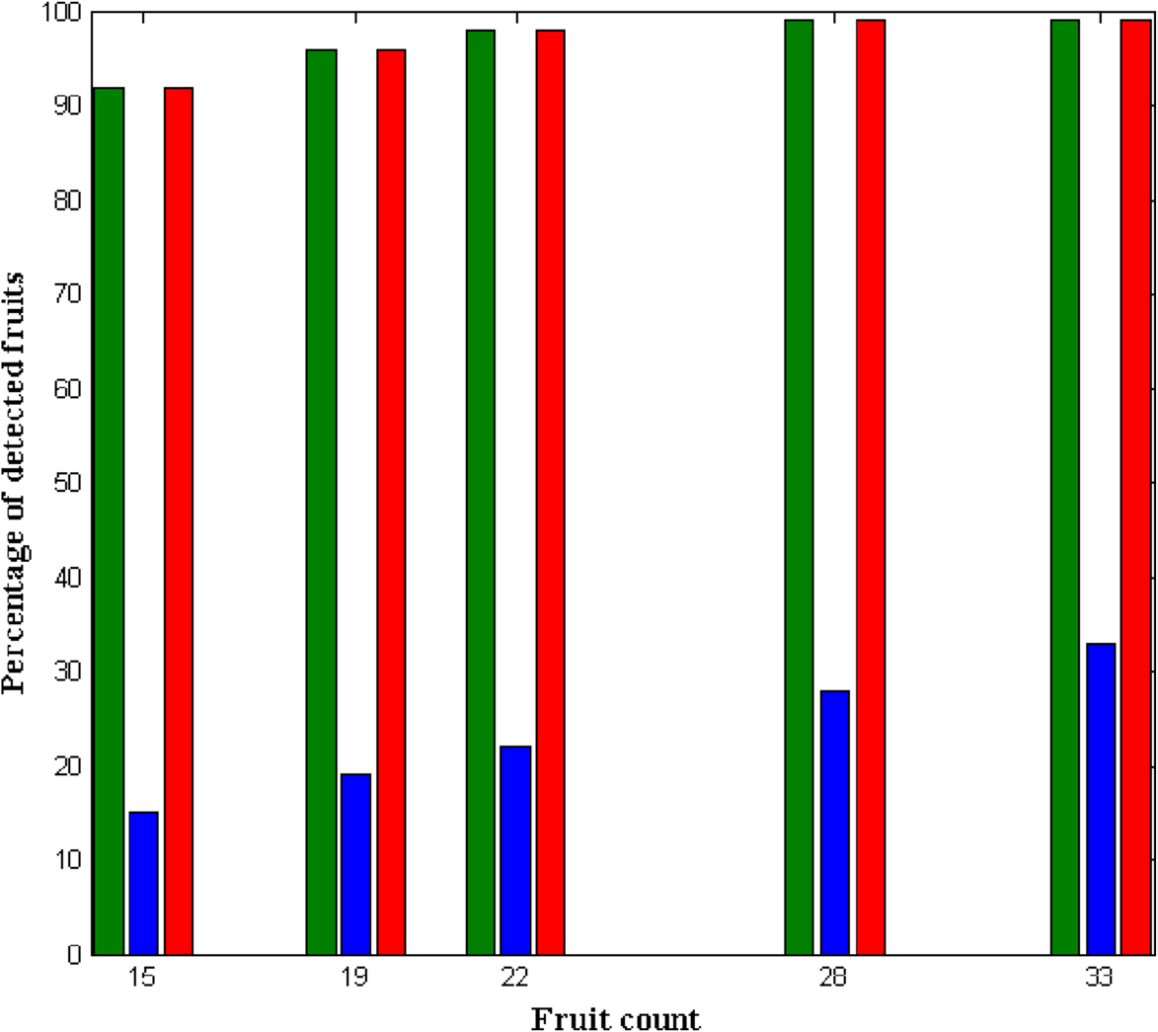
Fruit recognition and grasping estimation experiments: (**a**) only fruits in tree, (**b**) fruits with low ripe percent fruit and only low ripe percent fruit.

**Figure 8:**
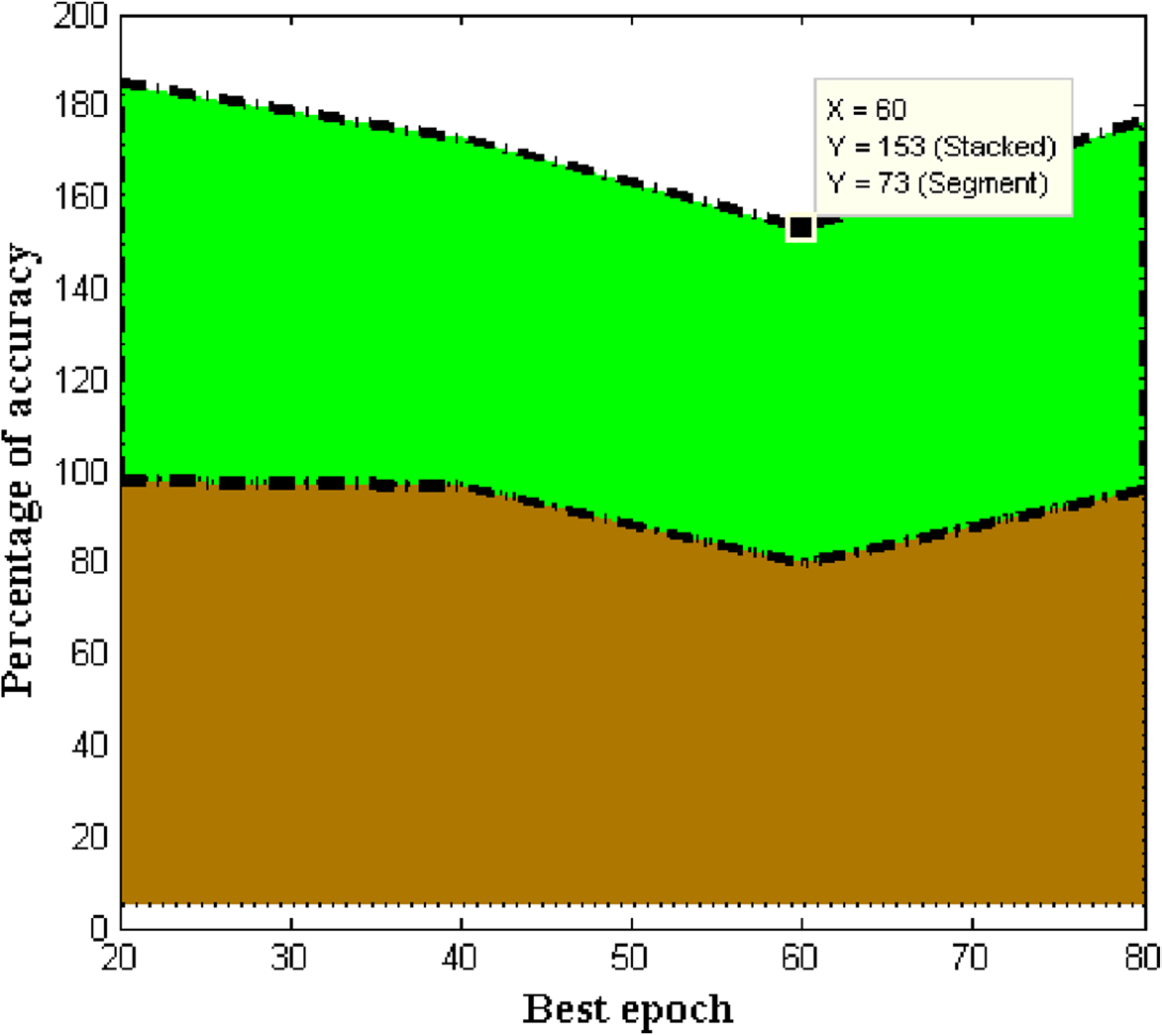
Comparison of accuracy using contours and open CV (existing vs proposed).

In this work, we used Open CV grabbing estimations to conduct fruit recognition and contouring on RGB-D photographs obtained in apple orchards as shown in Fig. 7. Fruit identification and segmentation were assessed using the F1 score and the IoU. The performance of Contours using Open CV with HSV grasping estimate, Open CV with HSV with Contours, and Open CV with HSV with Contours were compared and deliberated in Fig. 8. Estimation was more difficult in the orchard situations than it was in the interior environments. In this setting, the Open CV with HSV with Contours and Open CV with HSV with Contours fared significantly worse than in the indoor testing, while the Contours using Open CV grasping estimate did significantly better. Contours' IoU3D in the scenario of an orchard was 0.88, 0.76, and 0.78, respectively, using Open CV grasping estimate, Open CV with HSV with Contours, and Open CV with HSV with Contours.

Even when sensory input was inadequate, contours employing OpenCV gripping estimates worked effectively in determining grip orientation. Contours estimated using OpenCV grasping had a mean orientation estimate error of 6.6, which is in an acceptable limit.

### Case study 3

**Figure 9:**
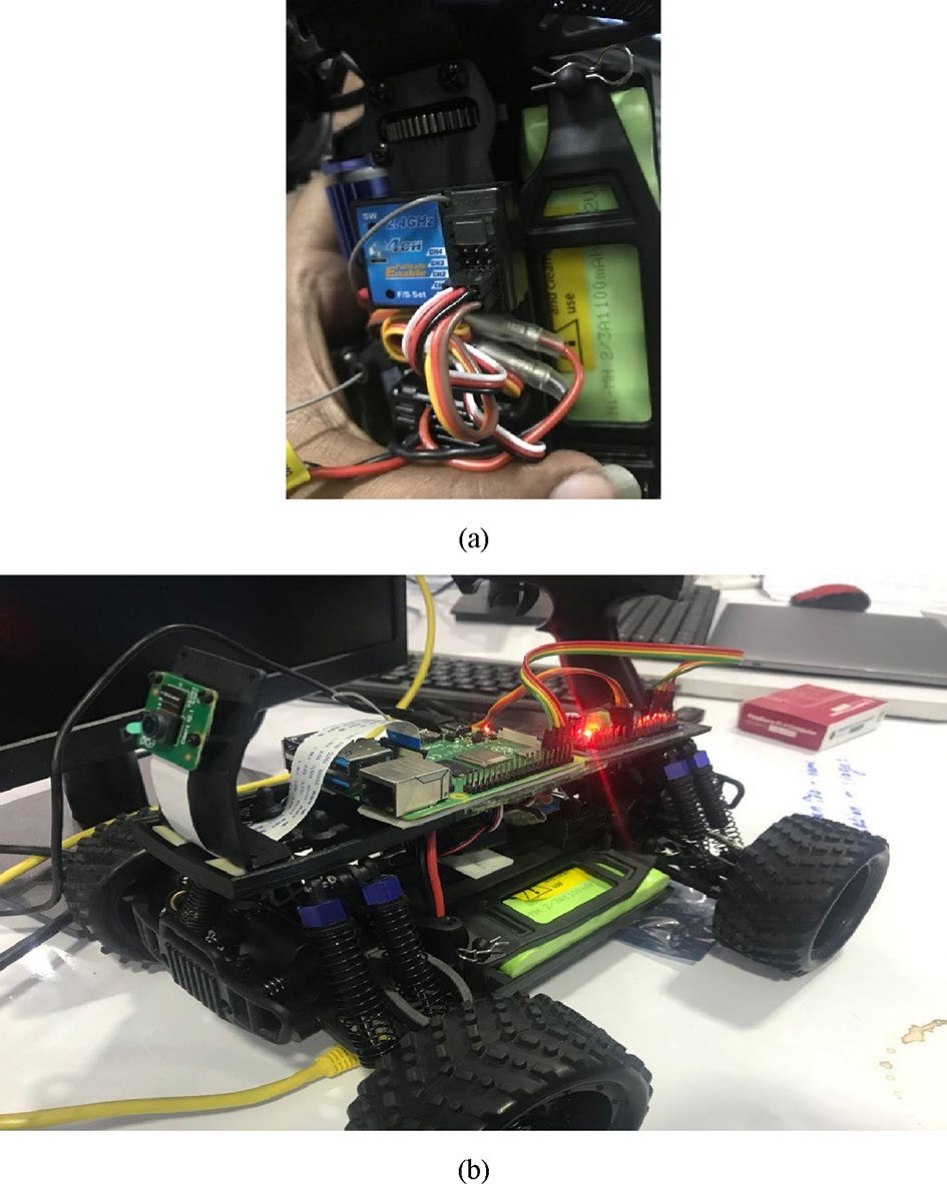
Experiment setup in indoor environment: (**a**) indoor connection segments, (**b**) designed robot.

**Figure 10:**
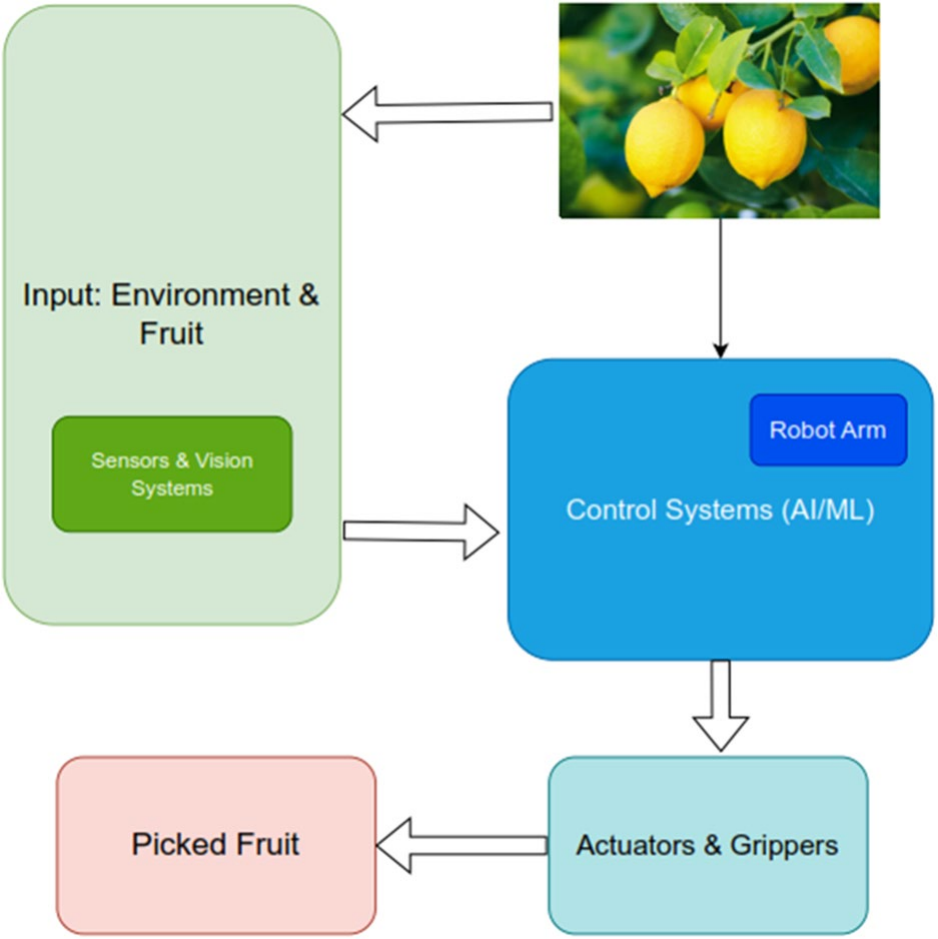
Experiment setup in outdoor environment with four phase considerations.

To determine the success rate of robotic harvesting, we randomly assigned the number, distribution, and placement of oranges on the orange tree. The experimental setup in indoor environment is illustrated in Fig. 9 and the four phases of the robotic grasping procedure are represented in Fig. 10 where the fruit harvesting technique and the Pose prediction enabled harvesting method were tested and contrasted, as indicated in Table 1. The natural harvesting approach was just removing fruit without regard for the gripping position of each fruit.

**Table 1:** Existing vs. proposed.

References	Method	Pros and cons
^19,20^	Threshold and circle fitting	High false detection rate and lack of reliability
^21,22^	Support vector machine (SVM)	Simple to implement, over fitting, and reduced detection accuracy
^23–25^	Digital twin (DT) and texture classifier	Complex in detection, more time consumption for training and testing
^26,27^	Faster recurrent neural network (RCNN)	High computational complexity, and lack of scalability
^28–30^	Semantic segmentation and VGG	Over fitting, computational burden, and high training and testing time
^31,32^	Adaptive threshold and fusion	Low detection rate, inaccurate, and lack of reliability
^33–35^	Single shot detection	Ineffective, and high false detection

The experimental setup for robotic system that is connected for identifying robotic harvesting focuses on indoor environments where fruits are identified by various colour units. Moreover to provide complete characterization the robotic design in separated in to four parts that are used for fruit verification, grasping, collection units and subsequent identification paths. Hence for the above mentioned functions it is necessary to use a detection system with high accurate camera that can able to identify all fruits without any discontinuity. Further it is possible to provide identification factor in a much easy way at outdoor environments due to the presence of bright features but in indoor environments additional identification units are added in such a way to provide grasping estimate of robots.

**Figure 11:**
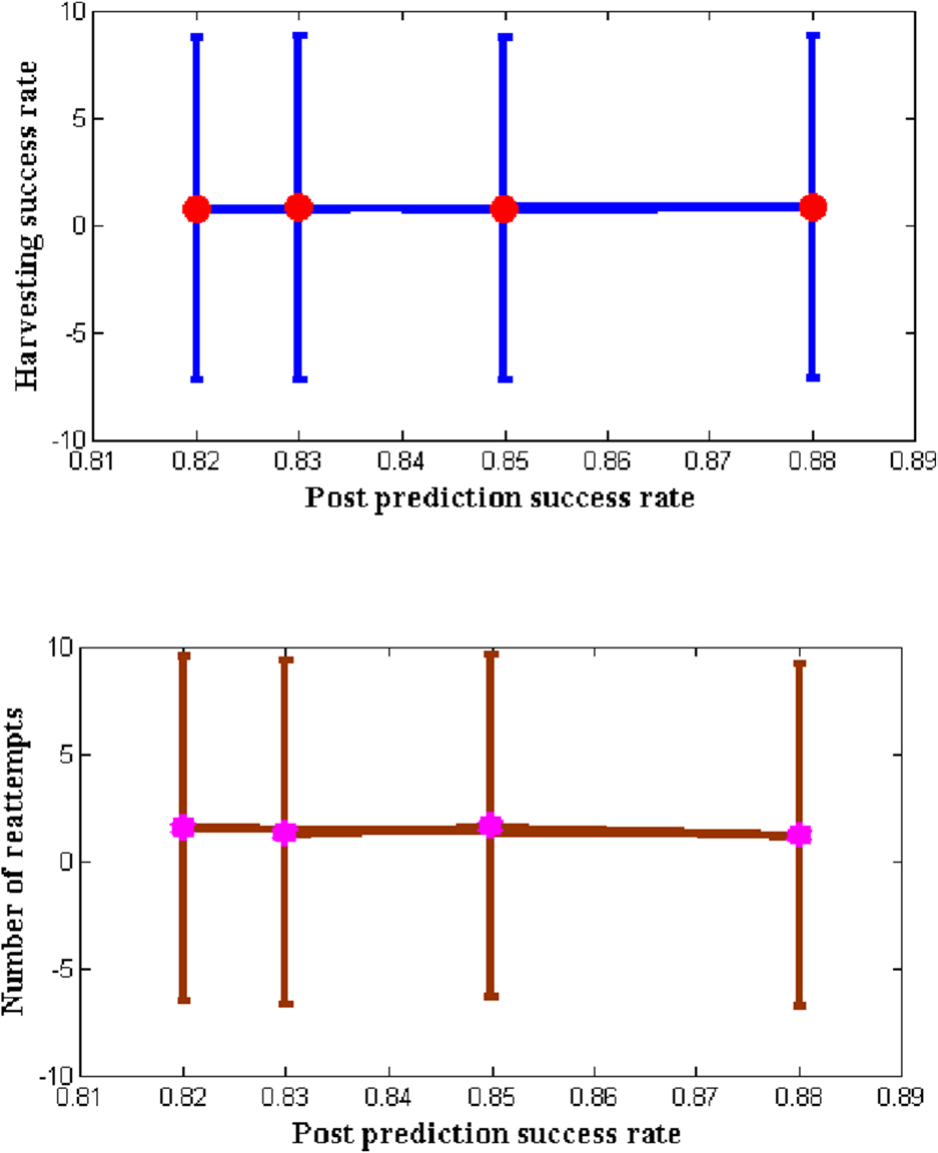
Success rate and number of reattempts for proposed and existing approach.

The accuracy of gripping position estimation was lower in both indoor and outdoor environments than on RGB-D picture data, as shown in Fig. 11. End-effector instability was found to be the cause of performance loss while the robotic arm was moving, which could lead to erroneous sensory input. From Fig. 11 it is observed that after harvesting the outcomes of robots are identified and plotted with respect to success rates and reattempts. In most of the grasping process that is completed by robotic actions it is realistic that success rate will be increased as compare to manual operation that is performed by individuals. The above mentioned situation is observed in the proposed method for fruit detections as the designed robot is having the capability to represent varying fruit type with proper identification procedures. Since the robot verifies the type of fruit before collecting it in carriers the number of reattempts to pick the same fruit is avoided in case of proposed method. However the above mentioned identification process is carried out only for indoor environments therefore in real time the considered identifications will change for outdoor environmental conditions. To verify this case study post prediction success rate is observed as 82, 83, 85 and 88% after collected units are counted therefore for increase in predictions rates the harvesting success rate is increased to 72, 73, 80 and 85% respectively. Similarly for the post predicted success rate the number of reattempts are identified to be minimized at it falls beyond 12%.

## Performance analysis

**Figure 12:**
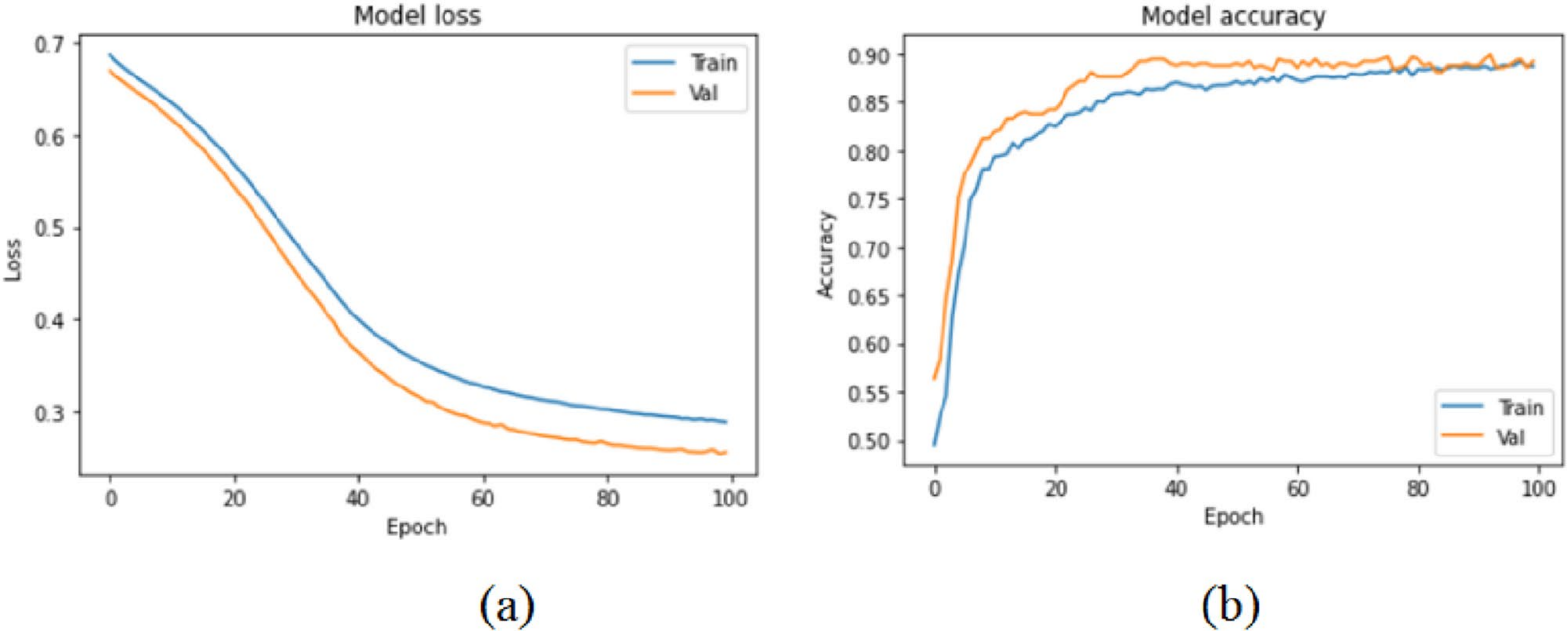
SSD architecture. (**a**) Model loss, (**b**) model accuracy.

As a result, following each robotic arm movement, we introduced a 0.5 s delay to verify that the sensory data input was correct. Gripping failure can be caused by a variety of factors, including a weak grip and a crowded environment. In the first case, one or two fingers of our modified three-fingered end-effector may not have connection with the target fruit, causing the target to slip off the gripper and fail, whereas in the second case, the gripper may have contact with the nearby fruit and led to frostbite for these neighbors. When compared to the naïve harvesting strategy, harvesting in accordance with the prediction of poses dramatically improved the efficiency of harvesting using robots and minimized the miss rates in indoor as well as outdoor contexts. Each trial took 4 and 6.5 s for the nave harvesting approach and Pose prediction assisted harvesting strategy, respectively. Overall, when tested in indoor as well as outdoor conditions, our suggested vision approach increased the accuracy and sturdiness of robotic harvesting systems as depicted in Fig. 12. The decrease in loss results in higher accuracy.

**Figure 13:**
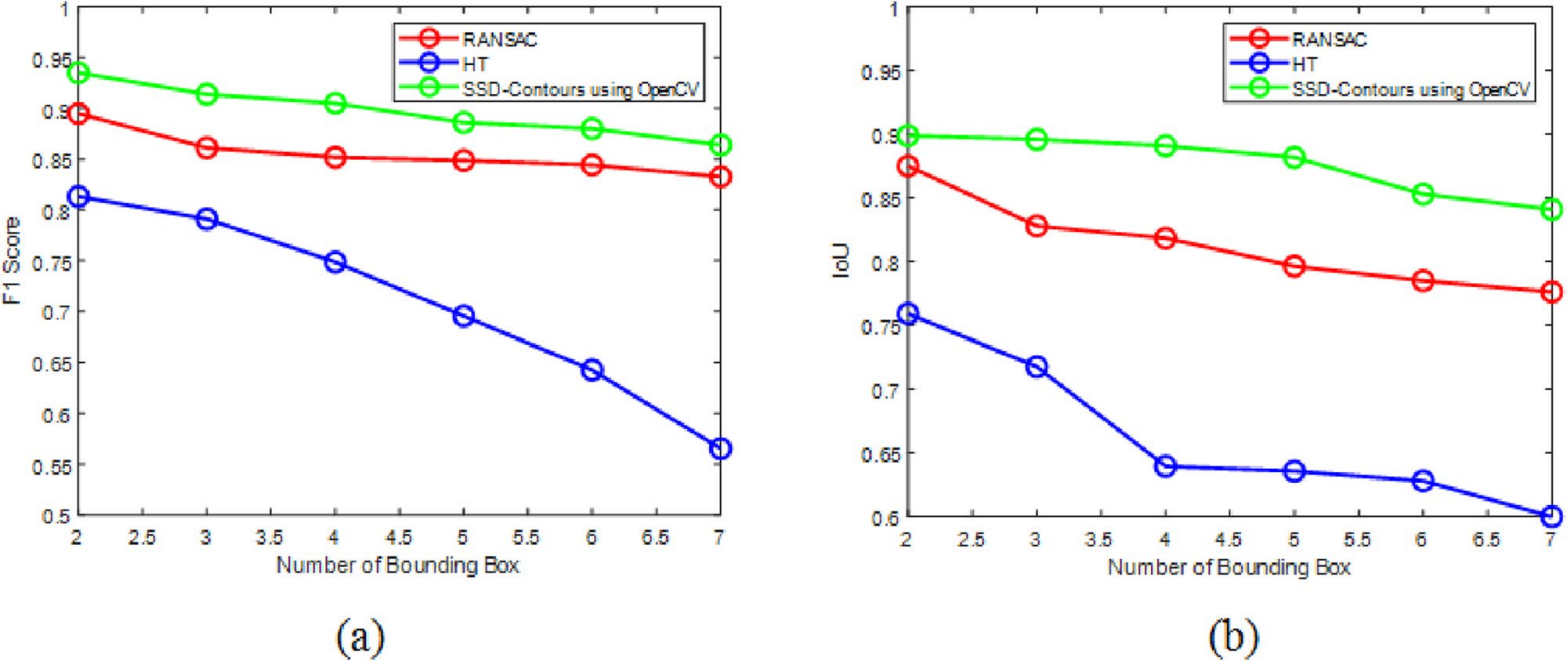
SSD contour (**a**) F1 score, (**b**) intersection over union (IoU).

**Figure 14:**
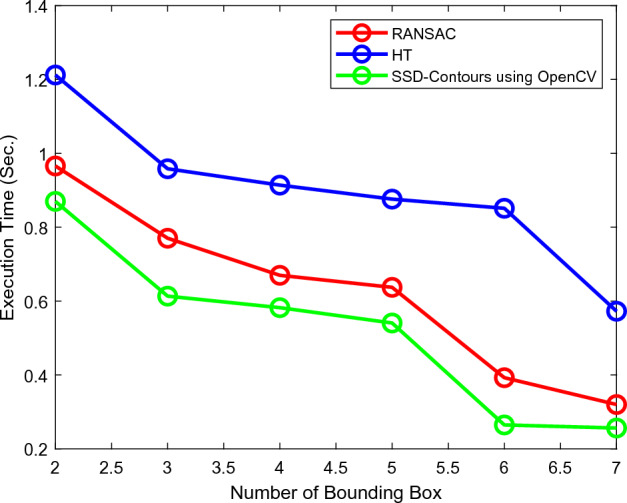
Execution time of SSD contours.

The recommended deep learning-based solution, Contours using Open CV, outperforms conventional techniques such as Open CV with HSV along with pose estimation and contours alone in the image data trials in terms of robustness when processing noisy data. This is because noise has a significant impact on the accuracy of the voting framework. Among the three techniques, our method had the greatest accuracy in detecting the fruit form estimation in dense clutter. Furthermore, the findings of the experiment showed that Contours using Open CV effectively and robustly predicted the approaching position while grasping under complicated situations including noise, outliers, and thick clutter. The experiment indicated that computer vision with posture estimation, HSV with contours, and computer vision with HSV can all perform When it comes to recognition of fruit and segmentation of the instances in orchard circumstances, it performs admirably. The proposed one-stage detector for fruit recognition has been proved to be accurate and computationally efficient. This light-weight computer vision SSD resulted in F1 score 0.94 as represented in Fig. 13. There call value is 0.826 and the value of accuracy is 0.9 on fruit detection. The Intersection over Union (IoU) which is a significant measure of accuracy. It indicates the overlap of the predicted and real bounding boxes. The SSD architecture is also efficient in accordance with the execution time its graphical representation as shown in Fig. 14.

Fruit recognition and segmentation activities are accomplished faster with this one-stage detector, resulting in a shorter average cycle time for fruit harvesting. In terms of future advances to the supplied approaches, the suggested Contours employing Open CV and contour functions might be merged into a single step. The robotic harvesting system's real-time performance is projected to increase with the achievement of fruit identification, segmentation, fruit modeling, and grabbing estimations in a single step. The inability to forecast the gripping position was mostly due to a defect in the test dataset sensory data. When the apple in the blue border box failed to generate a point cloud in the first place, the grasping approximation did not advance and was ruled a failure grasping estimation. Because there was no ground truth in this circumstance, there was no perfect value in the grabbing estimation. In this instance, contours using Open CV gripping estimates would always forecast a sphere with a short radius, which may be readily filtered out as outliers during execution.

**Figure 15:**
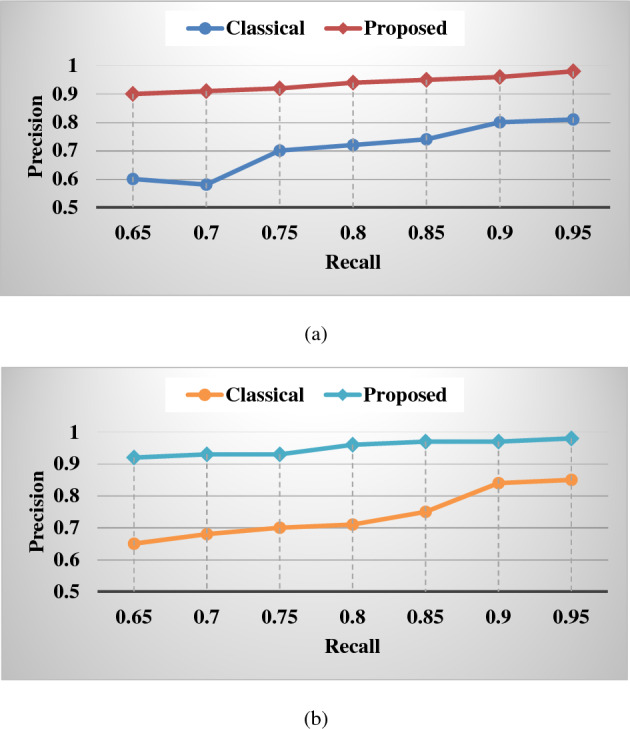
Detection rates. (**a**) Ripe fruit, (**b**) unripe fruit.

**Figure 16:**
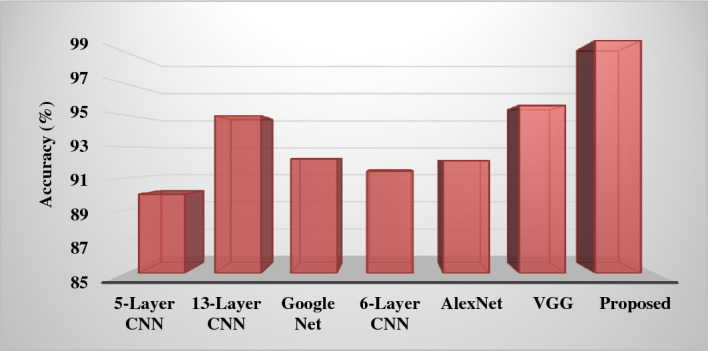
Accuracy of various fruit classification methodologies.

Even though the former was allowed by posture assessment and the latter can only interpret to the identified fruit, our proposed technique beat the naive harvesting method in terms of higher harvesting success rates and shorter re-attempt durations in robotic harvesting. Gripping failure can result from several circumstances, including a weak grip and heavy debris. In the first case, one or two of our modified three-fingered end-fingers effector's may lose touch with the target fruit (contact with neighboring branches instead or an incorrect gripping posture), causing the target to fall from the gripper and fail. In a congested setting, the gripper can easily contact nearby fruit, causing it to fall. When compared to the naïve harvesting strategy, harvesting based on predicting the pose dramatically improved the efficiency of robotic harvesting and minimized miss rate in indoor as well as outdoor situations.

4 $$mAP= \frac{1}{NC}\sum_{t=i}^{N}P(t)\Delta R(t)$$

where, $NC$ represents the number of categories, *N* is the count of thresholds, *t* indicates threshold, $P(t)$ is the precision, $R(t)$ is the recall, and $mAP$ denotes the average of multiple categories of area under the precision and recall values. The increased value of mAP states the improved performance of the classifier. According to the analysis, it is estimated that the proposed classification model overwhelms the other deep learning classifiers with improved mAP for the different types of fruits such as apple, mango and orange. Figure 15 shows the detection results of the classical CNN and proposed models with respect to the precision and recall values. Based on the analysis, it is observed that the detection results for both ripe and unripe fruit detection of the proposed model are highly improved, when compared to the classical CNN model. Consequently, the accuracy of several fruit classification methodologies are assessed as shown in Fig. 16 where the different architecture models of CNN classifier is considered for analysis. Overall, the obtained results indicate that the proposed deep learning based classification model overwhelms the existing architectures with improved fruit detection results.

Where, NC represents the number of categories, N is the count of thresholds, t indicates threshold, P(t) is the precision, R(t) is the recall, and mAP denotes the average of multiple categories of area under the precision and recall values. The increased value of mAP states the improved performance of the classifier. According to the analysis, it is estimated that the proposed classification model overwhelms the other deep learning classifiers with improved mAP for the different types of fruits such as apple, mango and orange. Figure 15 shows the detection results of the classical CNN and proposed models with respect to the precision and recall values. Based on the analysis, it is observed that the detection results for both ripe and unripe fruit detection of the proposed model are highly improved, when compared to the classical CNN model. Consequently, the accuracy of several fruit classification methodologies are assessed as shown in Fig. 16 where the different architecture models of CNN classifier is considered for analysis. Overall, the obtained results indicate that the proposed deep learning based classification model overwhelms the existing architectures with improved fruit detection results.

## Conclusions

This paper presents an experimental evaluation of a CNN-based robotic harvesting system designed to recognize and grasp fruits. The proposed methodology involves the utilization of a multi-functional network capable of simultaneously performing fruit identification and segmentation. Additionally, an SSD contours approach is employed, which utilizes a Convolutional Neural Network (CNN) based on Open CV, to analyze the point cloud data of the fruit. This analysis is used to estimate the grasping position for each fruit, thereby establishing the most optimal hold position. The adoption of a grasping stance is crucial when engaging in autonomous fruit picking activities. The fruit recognition network and SSD Contours, which have multiple functions, were trained and validated using RGB-D images obtained from both indoor and outdoor environments. The Open CV gripping estimate network was utilized in this process. The findings of the studies indicated that the proposed technique effectively facilitated accurate visual perception and grasping estimations. The computer vision Single Shot Multi Box Detector (SSD) attained F1 scores of 0.94, 0.826, and 0.9, along with recall and accuracy metrics for fruit identification. Additionally, the instance segmentation accuracy for fruit identification was measured at 0.82. Understanding estimates can be challenging. The Contours, employing Open CV grasping estimation, computer vision with posture estimation and HSV, and computer vision with the HSV algorithms, achieved IoU3D values of 0.88, 0.76, and 0.78, respectively, in the orchard scenario. The utilization of Open CV for contour detection outperforms the other two methods. The proposed robotic harvesting system underwent testing in both indoor and outdoor environments, demonstrating promising outcomes in terms of precision, resilience, and operational efficiency. The robotic harvesting system that has been designed demonstrates a harvest success rate of 0.75 and a cycle duration of 6.3 s.

## Data Availability

The datasets used and/or analysed during the current study are available from the corresponding author on request.
